# At Grammatical Faculty of Language, Flies Outsmart Men

**DOI:** 10.1371/journal.pone.0070284

**Published:** 2013-08-23

**Authors:** Ruedi Stoop, Patrick Nüesch, Ralph Lukas Stoop, Leonid A. Bunimovich

**Affiliations:** 1 Institute of Neuroinformatics, University and ETH of Zurich, Zurich, Switzerland; 2 School of Mathematics, Georgia Institute of Technology, Atlanta, Georgia, United States of America; University of Maribor, Slovenia

## Abstract

Using a symbolic dynamics and a surrogate data approach, we show that the language exhibited by common fruit flies *Drosophila* (*‘D.’*) during courtship is as grammatically complex as the most complex human-spoken modern languages. This finding emerges from the study of fifty high-speed courtship videos (generally of several minutes duration) that were visually frame-by-frame dissected into 37 fundamental behavioral elements. From the symbolic dynamics of these elements, the courtship-generating language was determined with extreme confidence (significance level > 0.95). The languages categorization in terms of position in Chomsky’s hierarchical language classification allows to compare *Drosophila*’s body language not only with computer’s compiler languages, but also with human-spoken languages. *Drosophila*’s body language emerges to be at least as powerful as the languages spoken by humans.

## Introduction

Over the centuries, the evolution of human language has been the subject of controversial discussions among philosophers, linguists and biologists. Yet, a consensus on what causes language to evolve has not been achieved. Traditionally, language has been thought of as a strictly culturally transmitted phenomenon, with few or no biological ties at all. In the second half of the 20th century, under Chomsky’s influence who considered that language is located in the brain and therefore is subject to biological conditions [1]–[3], this view started to change. A discussion arose what other driving forces of the evolution of language could be. As is any complex ability of humans or animals, language could be seen as the result of natural selection [4]. Chomsky suggested that the language grammar basics are hard-wired into the brain, and that this wiring may be the side-effect of the reorganization of the brain needed to cope with its growing size during evolution [5] (cf. [6] for a similar example). In order to study the evolution of language and to determine its driving forces, Chomsky and Schützenberger [7] proposed a hierarchical classification scheme, comprising grammars of increasing grammatical complexities: t-3 (left regular grammar) 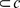 t-2 (context free grammar) 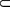 t-1 (context sensitive grammar) 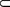 t-0 (Turing machine), able to account for the changes undergone. This classification approach has been used to compare spoken human languages, for distinguishing compiler languages, as a basis for the theory of automata, and for classifying dynamical systems [8]. In this paper, we apply this widely accepted classification scheme to the precopulatory dance of *Drosophila melanogaster*, where we show, by combining a nonlinear symbolic dynamics with a surrogate data analysis approach, that the dance of this fly is generally of complexity t-1: It is as, or even more, grammatically complex as the Dutch or the Swiss-German [9], the most complex spoken western languages (generally, human languages fall into Chomsky hierarchy t-2 [9], [10]). Note that our general approach could also be applied to other taxonometries of language characterization.

In the animal world, courtship ranges from simple rituals to complex communication-like behaviors. Despite its high cost for the animal (energy- and death toll-wise), the origins and purpose of courtship are still not well understood. A natural hypothesis is that courtship is an evolutionary optimization mechanism that a species may or may not take advantage of. Behavior is characterized by rituals that consist of well-chosen sequences of individual actions. Since it is in the nature of these rituals that they need to be repeated if required, we characterize behavior by sequences of indecomposable closed cycles of indecomposable individual actions, so-called irreducible cycles of irreducible acts [11], [12]. This approach is also motivated by the theory of complex dynamical systems, where it has been shown that such systems can be reduced to a minimal set of closed sequences of actions (there called ‘irreducible closed orbits’). From this set the system can systematically be approximated by combining ever more of these sequences, starting with the shortest ones (for detailed references cf. Ref. 12). Living in a simple and evolutionarily fast environment, *D.* provides a well-suited courtship behavior testing case. Using a decomposition of courtship data into irreducible cycles, it has been found that with high confidence during *D.*’s precopulatory courtship, individual information is transmitted to the prospective partner, i.e. genuine communication with essential information exchange takes place [11], [12] (Fig. S2).

**Figure 1:**
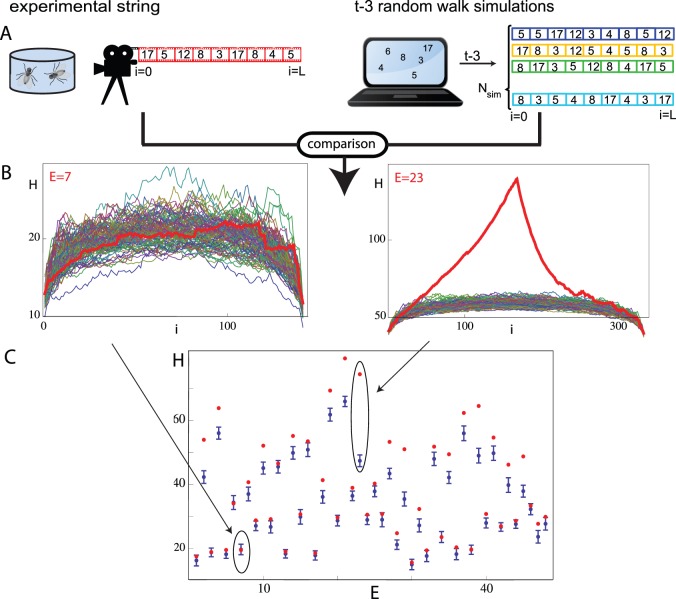
Comparison of experimental files to grammar-generated files. A) For each experimental symbol string, strings with the identical symbol probabilities are generated using a t-3 random walk. B) For each string (observed and simulated), 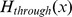 is calculated. Thick red lines: experiments, thin lines: t-3 random walks. 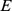 numbers the experiment. C) Red dots: 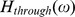 for the experimental files. Blue dots: Mean values of 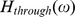 from 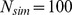 t-3 random walks. Bars: One standard deviation. For two thirds of all files, the t-3 model fails.

To the best of our knowledge we use here for the first time Chomsky’s classification scheme to characterize courtship and animal body language. Although the question by what grammar a given experimental data was generated is in its narrower sense undecidable (the ‘string problem’ is undecidable [13]), we are able to provide an answer in the statistical sense: Namely, we show that it is very unlikely that *D.*’s body language is generated by grammars of complexity lower than those of human languages. For some data we find indications of a t-1 grammar underlying their generation, which reaches beyond the grammatical complexity of human language. An overview of the experimental and computational procedures is presented in Fig. 1.

## Materials and Methods

The data that we use in this study originates from experiments where the courtship behavior of a pair of fruit flies is recorded in an observation chamber at fixed environmental conditions of 25°C and 75% humidity. From high-speed camera recordings of 30 frames per second, we isolated 37 fundamental behavioral acts and coded the recordings accordingly [11] (Fig. S1). Fundamental acts are body movements that can freely be combined with each other. Besides pairing single normal females in the immature, mature and mated states with single normal males, additionally fruitless mutant males [11] were paired with either mature females or with mature normal males, leading to five types of experiments. Since either of the protagonists gives rise to a time series, ten classes of experimental time series were obtained in this way. Tagging each fundamental act by an integer number, each camera episode is represented by a string or time series of these symbols. A mature female as the protagonist in the presence of a normal male, e.g., generates in this way a time series as
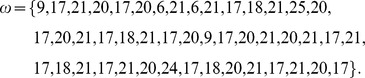



The simplest grammatical model for the putative generation of the experimental time series is a t-3 grammar from the Chomsky hierarchy of languages. This model is equivalent to a random walk on the given set of symbols with probabilities given by the symbol frequencies observed in the respective experiments, but with no further restrictions imposed. If *D.*’s body language is of low complexity, the observed strings should fit well into the random walk model. From simulating the random walk based on the observed symbol probabilities of each experiment, we obtained from each experimental file a set of surrogate files to compare with (Fig. 1A; throughout our investigations, we use 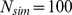 simulated random walks). For the comparison, a figure of merit is used. Every time series 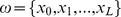 is characterized by products along the string of the probabilities 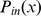 - measuring that a random walk starting at 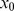 ends at point 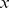 - with 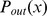 measuring the probability that a random walk starting at 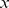 reaches point 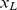.

For the unrestricted random walk, these probabilities are
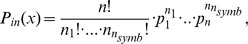


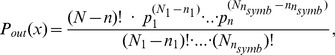
where 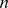 is the number of steps needed to reach point 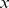, producing 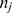 repetitions of the symbol tagged with index 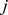.

The entropy 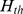 associated with a string realization is based on the local walk-through probability 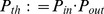, evaluated along the string, as
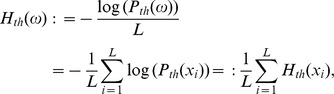
with 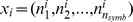 the coordinate of point 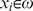 in the symbol space. In the figures, 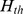 will always be abbreviated by 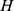 (Fig. 1B).

## Results: Courtship Language Classification

We evaluated 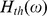 for each experiment and for the corresponding surrogate random walks. For the latter, we also determined the mean values and the standard deviations (Fig. 1C). Whereas the t-3 model generates strings with similar 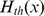 characteristics for approximately one third of the experimental data (Fig. 1B, left panel) for the remaining two thirds, this description fails (Fig. 1B, right panel). In the latter cases, the experimental 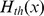 dramatically differs from those obtained for the t-3 model: The experiment’s clear peak around position 170 is very unlikely to be reproduced by a simple random walk. The pyramid-like shape with its clear maximum of 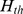 suggests that in the data, an eminent change has occurred in the way of how symbols are chosen from the alphabet.

**Figure 2:**
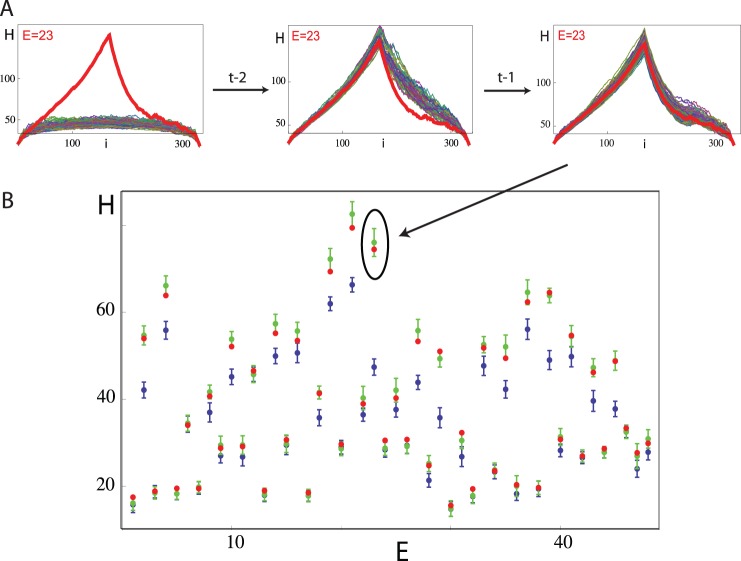
Example of an experiment that requires a t-1 random walk (E = 23). A) Improvement of the modeling by going from t-3 via t-2 to t-1 random walks. B) 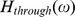 for each experiment and its surrogate set. Red dots: experiments. Blue: dots: mean values of 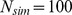 t-3 random walks; bars: one standard deviation. Green: mean values of 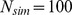 t-3, t-2, t-1 random walks; bars: one standard deviation. Some blue dots and bars are obscured by red and green dots and green bars. One can clearly see that the green dots approximate the experimental red dots much better than the blue dots.

To proceed with those experiments that do not fit into a t-3 model, we apply a recursive approach (‘t-3, t-2, t-1 model’). We split a string at the point of maximum 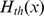, and model the partial strings 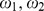 separately by corresponding random walks. Strings of the form 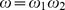 are generated from a t-2 (i.e. context-free) grammar, since a word 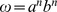, 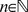 cannot be created by a t-3 grammar. t-2 grammars reproduce the characteristics of five of our experiments, they remain, however, to be inappropriate for about half of the data. The obvious solution then is to expand the latter into ever more partial walks. Technically, for each file we simulate a set of 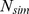 random walks. On this set, we calculate 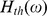, their average and their standard deviation. If the original file’s 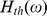 falls within a standard deviation from the computed average, the random walk describes the string well and the string is considered to be t-3. Otherwise, by splitting the string 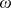 at the maximum of 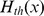, we obtain 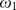 and 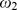. For these partial strings, random walks are then performed separately, and compared to the original data. If they are close enough in the above sense, we consider the string to be t-2. Otherwise we proceed recursively, which implies context-sensitivity [13] and therefore a t-1 grammar (Fig. 2A.). Surrogate walks generated according to this ‘t-3, t-2, t-1′-construction (Fig. 2B, green dots and intervals), now perfectly capture the experimental data.

**Figure 3:**
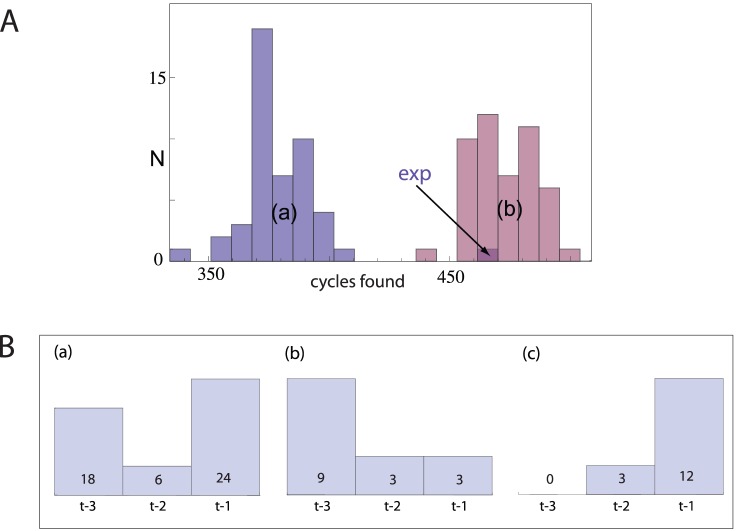
Effect of random-walk grammar on closed cycles. A) Histogram of the cumulative number of closed cycles across all data files from a) in a t-3 random walk model of the data, b) in t-3, t-2, t-1 random walk model according to the data’s classification,. The experimental data (“exp”) with 468 cycles fits well only into the t-3, t-2, t-1 model. Histograms are based on 100 simulations for each experimental file. B) Distribution of t-3, of t-2, of t-1 classifications a) across all experiments, b) across all experiments with females, c) across all experiments with normal males, where the absolute numbers are exhibited.

A key characteristic for assessing behavior - and therefore also the underlying generative grammar - is by irreducible cycles [11], [12]. We observed in surrogates from files that we classified as t-2 or t-1, a massive increased number of irreducible closed cycles (Fig. 3A). Whereas our experimental data clearly stands out from a surrogate distribution based on t-3 grammar models for all files (Fig. 3Aa), it fits well into the surrogate distribution based on the ‘t-3, t-2, t-1’-model (Fig. 3Ab. The numbers of cycles deviate from the cumulative numbers of different cycles exhibited in ref. 2, but demonstrate the same tendency). This not only corroborates our conclusion that a higher-complex grammar underlies *D.*’s body language, it also validates the relevance of this observation for the behavioral context.

## Discussion

The comparison between the behavior of all observed female flies and all observed normal males reveals that female flies tend to follow t-3 or t-2 grammars, while normal males tend to use t-1 (Fig. 3B). This provides a novel insight into the role of the courtship protagonists depending on their sexual group from the grammatical perspective. More fundamentally, we stress the conclusion that *D.*’s precopulatory body language is not the result of the simplest grammar t-3 (i.e., a random walk on states of a finite automaton). There is a general agreement that natural human languages fall mostly into t-2 Chomsky’s characterization (with among the European languages the Swiss-German and the Dutch showing the highest degree of grammatical complexity [9]). On the basis of our analysis one can safely say that the *D.*’s body language is of no lesser grammatical complexity than the spoken language of humans.

The supremacy of human intellect can thus not be founded in the formal grammatical complexity of the language used. Surprisingly, species as simple as the fruit fly have recursive elements too (recursion is often the key argument for distinguishing between t-3 and t-2 grammars [14]). It appears, however, that only humans have acquired a kind of awareness of theses structures and have learnt to purposefully use them. It is conceivable that the evolutionary anatomical changes of our growing neocortex has led to an outsourcing of loops and stacks to other areas of the brain, which may have brought along a notion of loop-awareness. The usefulness of this concept (for navigation tasks or for general counting processes), may have enhanced the awareness and the purposeful use of these structures further during evolution. This in distinction to animals that in principle do have these structures as well (as the *D.* example), but are not aware of them and do not use them purposefully. An indication of the importance of infinite loops [15] can be seen in how they particularly fascinate children, as exemplified by the children rhyme: “Once, there was a man with a hollow tooth, and in this tooth there was a little box, and in this box there was a paper, on which was written: Once, there was a man.” (translation from Swiss-German). It is hard to imagine an animal finding such a construct as fascinating as we do.

## Supporting Information

Figure S1 **Table of** ***Drosophila*** **acts.**
(PDF)

Figure S2 ***Drosophila*** **experimental group identification based on closed-orbit characterization of behavior.** A) mean-difference test at p-value 0.9 for whether observed similarities across all the population could origin from the same distribution (white: yes, black: no); B) individual similarity (white: low, black: high). Group pooling can be justified by means of majority voting.
(PDF)
