# Supplementary material for: At Grammatical Faculty of Language, Flies Outsmart Men
Source: PLoS One. 2013 Aug 23;8(8):e70284. doi: 10.1371/journal.pone.0070284 (PMC3751931; doi:10.1371/journal.pone.0070284)
Supplement: Figure S1 — Table of Drosophila acts. (PDF) [file pone.0070284.s001.pdf]

	Drosophila acts	Gender
1	Abdobend	Female
2	Abdotwist	Female
3	Attemptcop	Male
4	Circling	Male
5	Copulation	Male
6	Decamp	Male/female
7	Fencing	Male
8	Following	Male
9	Grooming forelegs	Male/female
10	Grooming hindlegs	Male/female
11	Headpos	Male/female
12	Kick hindlegs	Female
13	Licking	Male
14	Orientation	Male
15	Ovipext	Female
16	Run	Male/female
17	Standing	Male/female
18	Still	Male/female
19	Tapping	Male
20	Walk left	Male/female
21	Walk right	Male/female
22	Wingext left	Male
23	Wingext right	Male
24	Wingflicks left	Male/female
25	Wingflicks right	Male/female
26	Wingflutter	Female
27	Wingspread	Male/female
28	Wingwave	Male
29	Wingflicks unspec.	Male/female
30	Grooming midlegs	Male/female
31	Tapping forelegs	Male
32	Kick midlegs	Female
33	Walk unspec.	Male/female
34	Kick unspec.	Male/female
35	Wingflap	Male/female
36	Run right	Male/female
37	Run left	Male/female
