# Supplementary figures and images for: At Grammatical Faculty of Language, Flies Outsmart Men
Source: PLoS One. 2013 Aug 23;8(8):e70284. doi: 10.1371/journal.pone.0070284 (PMC3751931; doi:10.1371/journal.pone.0070284)

(a)

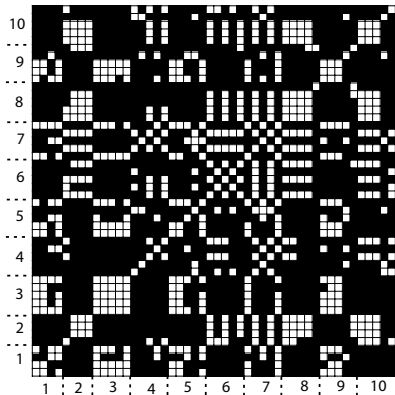

(b)

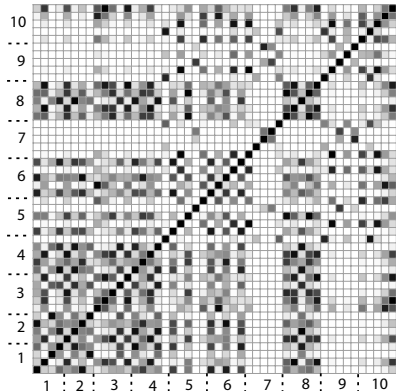

Supplement: Figure S2 — Drosophila experimental group identification based on closed-orbit characterization of behavior. A) mean-difference test at p-value 0.9 for whether observed similarities across all the population could origin from the same distribution (white: yes, black: no); B) individual similarity (white: low, black: high). Group pooling can be justified by means of majority voting. (PDF) [file pone.0070284.s002.pdf]
